# Enu Mutagenesis Identifies a Novel Platelet Phenotype in a Loss-Of-Function Jak2 Allele

**DOI:** 10.1371/journal.pone.0075472

**Published:** 2013-09-25

**Authors:** Nicole M. Anderson, Mojib Javadi, Elizabeth Berndl, Zorana Berberovic, Monica L. Bailey, Kai Huang, Ann M. Flenniken, Lucy R. Osborne, S. Lee Adamson, Janet Rossant, Christin Carter-Su, Chen Wang, Kelly M. McNagny, Robert F. Paulson, Mark D. Minden, William L. Stanford, Dwayne L. Barber

**Affiliations:** 1 Institute of Medical Science, Faculty of Medicine, University of Toronto, Toronto, Ontario, Canada; 2 Department of Medical Biophysics, Faculty of Medicine, University of Toronto, Toronto, Ontario, Canada; 3 Institute of Biomaterials and Biomedical Engineering, Faculty of Medicine, University of Toronto, Toronto, Ontario, Canada; 4 Department of Obstetrics and Gynecology, Faculty of Medicine, University of Toronto, Toronto, Ontario, Canada; 5 Department of Medicine, Faculty of Medicine, University of Toronto, Toronto, Ontario, Canada; 6 Department of Molecular Genetics, Faculty of Medicine, University of Toronto, Toronto, Ontario, Canada; 7 Samuel Lunenfeld Research Institute, Toronto, Ontario, Canada; 8 Mount Sinai Hospital, Toronto, Ontario, Canada; 9 Toronto Centre for Phenogenomics, Toronto, Ontario, Canada; 10 Hospital for Sick Children, Toronto, Ontario, Canada; 11 Department of Molecular and Integrative Physiology, University of Michigan, Ann Arbor, Michigan, United States of America; 12 The Biomedical Research Centre, University of British Columbia, Vancouver, British Columbia, Canada; 13 Department of Veterinary and Biomedical Sciences, Pennsylvania State University, University Park, Pennsylvania, United States of America; 14 Ontario Cancer Institute, Toronto, Ontario, Canada; 15 Sprott Centre for Stem Cell Research, Ottawa Hospital Research Institute, Ottawa, Ontario, Canada; 16 Department of Cellular and Molecular Medicine, University of Ottawa, Ottawa, Ontario, Canada; University of Leuven, Belgium

## Abstract

Utilizing ENU mutagenesis, we identified a mutant mouse with elevated platelets. Genetic mapping localized the mutation to an interval on chromosome 19 that encodes the Jak2 tyrosine kinase. We identified a A3056T mutation resulting in a premature stop codon within exon 19 of Jak2 (*Jak2* ^*K915X*^), resulting in a protein truncation and functionally inactive enzyme. This novel platelet phenotype was also observed in mice bearing a hemizygous targeted disruption of the Jak2 locus (*Jak2* ^*+/-*^). Timed pregnancy experiments revealed that *Jak2* ^*K915X/K915X*^ and *Jak2* ^*-/-*^ displayed embryonic lethality; however, *Jak2* ^*K915X/K915X*^ embryos were viable an additional two days compared to *Jak2* ^*-/-*^ embryos. Our data suggest that perturbing JAK2 activation may have unexpected consequences in elevation of platelet number and correspondingly, important implications for treatment of hematological disorders with constitutive Jak2 activity.

## Introduction

Cytokines play an integral role in hematopoiesis by providing growth signals to progenitor and committed cells that promote mitogenesis, survival and in some cases differentiation. Once bound to their cognate receptors, cytokines mediate downstream signaling through activation of components of the Jak-Stat signaling pathway. Thrombopoietin (Tpo) is the principal cytokine regulator of megakaryopoiesis, through binding to its cognate receptor Mpl. Tpo activates the Jak2 and Tyk2 tyrosine kinases [1] as well as the Stat3 and Stat5 transcription factors [2,3,4]. The importance of Tpo, its receptor and proximal signaling pathways in platelet function is illustrated by the discovery of gain-of-function mutations in Tpo [5], Mpl [6,7] and Jak2 [8,9,10,11] that all result in Essential Thrombocythemia (ET). Similarly, loss-of-function mutations in Mpl have been documented in Congenital Amegakaryocytic Thrombocytopenia [12,13]. Jak2 is critical for murine embryogenesis as mice lacking Jak2 expression die of anemia at E12.5 [14,15].

While screening ENU mutagenized mice for dominant hematopoietic defects, we identified a mouse with thrombocythemia and determined that the mutation resulted in a truncated allele of Jak2 that lacked catalytic activity. Analysis of this mutation has uncovered a novel function of Jak2 in the megakaryocyte/platelet lineage.

## Materials and Methods

### Mice and ENU mutagenesis

*C57Bl6/J* (*B6*) and *129S1/SvImJ* (129) mice were purchased from The Jackson Laboratory. *Jak2* ^*+/-*^ mice (on the B6 genetic background) were provided by Dr. James Ihle, Memphis, TN. All mice were maintained in specific-pathogen free facilities at the Toronto Centre for Phenogenomics or Ontario Cancer Institute. Animal protocols were approved by the OCI Animal Care Committee (Permit Number 1517). All efforts were made to reduce animal suffering.

To induce random mutations, one intraperitoneal injection of 150mg/kg ENU was administered to male *129* mice (mutagenized strain) [16]. The F1 generation (*129*;*B6*) was produced by out-crossing ENU-mutagenized males to *B6* (mapping strain) females – pups from this breeding were designated generation 1 (G1). G1 mice were screened to detect dominant traits deviating from normal homeostatic venous blood parameters by at least two standard deviations from ‘normal’ G1 parameters. Affected mice with elevated platelets were sequentially back-crossed to *B6* mice for genetic mapping. The *Jak2* ^*K915X*^ allele was maintained on a *B6* background by intercrossing heterozygous or wild type (WT) mice. Timed matings were performed on G9 animals and peripheral blood analysis was completed on G10 mice.

### Hematologic analysis, genetic mapping and sequencing

Peripheral blood from 6-8 week old mice was collected by saphenous venipuncture. Complete blood counts (CBC) were performed using a Coulter Ac-T Differential Hematology Analyzer. *Jak2* ^*+/+*^ mice were littermate controls of *Jak2* ^*+/-*^ animals. *Jak2* ^*Control*^ mice are littermate controls of *Jak2* ^*K915X*^ G10 breedings. Bone marrow sections were prepared from femurs of 12-week old mice. Femurs were fixed in 10% formaldehyde and then sectioned (4 µm) and stained with Hematoxylin and Eosin (H&E) at the CMHD pathology core (http://www.cmhd.ca/enu_mutagenesis/pathology.html). Affected mice were sequentially bred to *B6* to confirm heritability and to genetically map the mutation using microsatellite base genome scan and single-nucleotide polymorphism markers differentiating *129* and *B6* alleles [16]. Once the mutation was mapped to a 6.7Mb region of chromosome 19, candidate gene analysis was used to select genes for exon sequencing [14,15].

### Genotyping

Multiplex PCR was used to genotype *Jak2* ^*K915X*^ and *Jak2* ^*+/-*^ mice using genomic DNA prepared from tail or biopsy tissue [14]. All Jak2K915X genotyping was performed at The Centre for Applied Genomics using a custom TaqMan SNP genotyping assay. The custom assay was used to discriminate between the wild type allele (A 3056nt) and the Jak2^K915X^ allele (T 3056nt).

### Clonogenic assays

CFU-C, CFU-E and CFU-Mk assays were performed as previously described [17,18].

### 5-fluorouracil and Phenylhydrazine Priming

Six to eight-week old mice were injected with 5-fluorouracil (5FU) or Phenylhydrazine (PHZ), as previously described [18,19]. Briefly, 5FU was administered at 120 µg/kg and blood was collected at Days 0, 6, 8 and 13. PHZ was delivered by intraperitoneal injection at 100 µg/kg and peripheral blood was harvested at Days 0, 1, 7 and 9. Complete blood counts were performed with a HEMAVET 950 (Drew Scientific Inc.).

### Transfection and Cell Culture

293T cells (ATCC) were transfected with HA-tagged Jak2 or HA-Jak2 K915X. Alternatively, Jak2 constructs were generated that expressed the 3’ UTR or had the 3’ UTR removed. Thirty-six hr after transfection, cells were washed, lysed as described [20]. Lysate fractions were resolved via SDS-PAGE and transferred to PVDF membranes for Western blotting experiments.

### Western blotting

Membranes were blocked in optimal blocking agent (either 2.5% bovine serum albumin or 5% skim milk powder in 50 mM TrisHCl (pH 8.0), 150 mM NaCl, 0.1% Tween 20 (TBST)) for 1 hr. Primary antibody incubations were performed for 1 hr, followed by 30 min washing in TBST. Secondary incubations were performed with HRP-Sheep anti-mouse IgG (GE Healthcare Life Sciences, Mississauga, ON) or HRP-Protein A (GE Healthcare Life Sciences, Mississauga, ON) for 30 min. After washing, membranes were developed by ECL.

### Antibodies

Anti-Jak2 and β-tubulin antibodies were purchased from Cell Signaling Technology (Beverley, MA) and Millipore (Billerica, MA). The HA antibody was from Covance (Laval, QC). Phosphorylation-specific pSer-523 [21] and pTyr-570 [22] Jak2 antibodies have been previously characterized.

### Microscopy

Megakaryopoiesis was assessed by microscopic examination of bone marrow of femurs on histology sections. Megakaryocytes are identified by their characteristic morphology of large size, lobulated nuclei and abundant cytoplasm and quantified by counting the number of megakaryocytes per microscopic field under a 40x objective.

## Results

**Figure 1:**
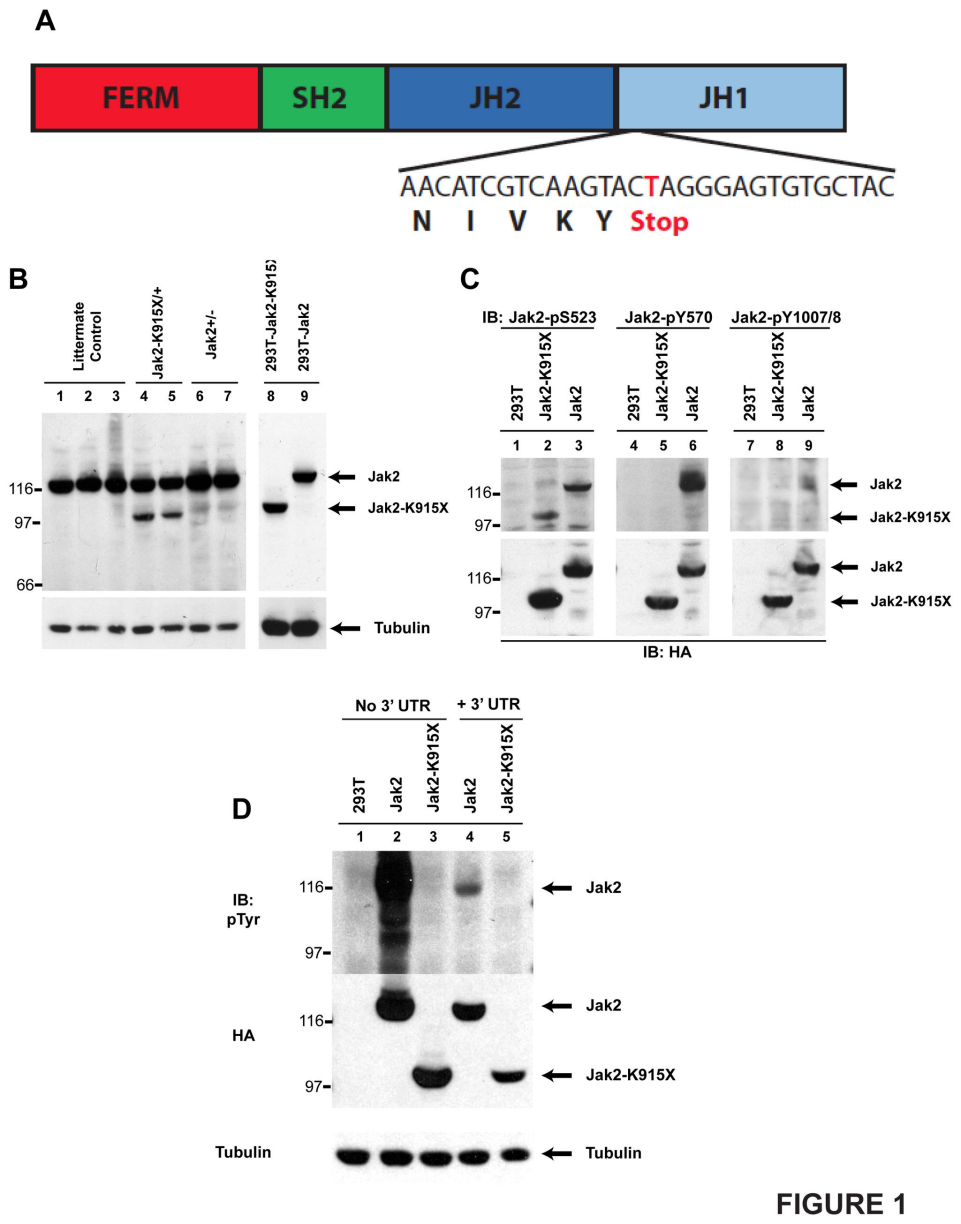
Strain 7254 is defined by a K915X mutation and generates a non-functional truncated Jak2 protein. (A) The Jak2 domain structure is indicated. The DNA sequence from *7254* splenocytes and corresponding protein sequence are also shown. (B) Splenocytes were harvested from phenylhydrazine primed *Jak2* ^*K915X*^ and *Jak2* ^*+/-*^ mice and their wild type littermates. HA-tagged Jak2 and Jak2^K915X^ were also expressed in 293T cells. A Western blot was performed with a peptide-specific JAK2 antibody. (C) 293T cells were transfected with cDNAs encoding HA-Jak2 or HA-Jak2 K915X. Western blotting was performed with phosphorylation-specific antibodies that recognize pSer-523, pTyr-570 and pTyr-1007/1008 in Jak2. The membranes were stripped and reprobed with an anti-HA antibody. (D) HA-tagged versions of Jak2 and Jak2^K915X^ with or without the JAK2 3’ UTR were expressed in 293T cells. Western blots were performed with 4G10 anti-phosphotyrosine and HA antibodies. Immunoblotting with anti β-tubulin was performed to demonstrate equal loading.

We identified a G1 mouse, strain *7254*, with elevated platelets. Back-crossing on to the B6 strain and SNP-based mapping resulted in the identification of a 6.7 Mb heritable region on chromosome 19 as the interval encoding the responsible mutation. We noted that *Jak2* was within this interval and hypothesized that the mutation underlying the *7254* phenotype may be in *Jak2*. We performed genomic DNA sequencing which identified an A3056T mutation in exon 19 of the Jak2 locus. This mutation leads to a K915X premature stop codon in the functional JH1 kinase domain of Jak2 (Figure 1A).

Western blotting of splenocytes isolated from *Jak2* ^*K915X*^ *, Jak2* ^*+/-*^ and *Jak2* ^*+/+*^ mice revealed a novel, truncated 95 kDa protein in *Jak2* ^*K915X*^ mice (Figure 1B, lanes 4 and 5), that co-migrated with the expressed Jak2^K915X^ protein in 293T cells (lanes 8 and 9).

Recent evidence has suggested that the Jak2 JH2 domain possesses weak intrinsic kinase activity [23,24]. Considering that the JAK2 K915X mutation resides in the Jak2 JH1 domain, we tested whether JAK2 K915X protein product is catalytically active. Both wild type Jak2 and Jak2 K915X are phosphorylated at Ser-523 [21] (Figure 1C, lanes 2 and 3). However, only wild type Jak2 is phosphorylated at Tyr-570 [22] (lane 6) and Tyr-1007/1008 (lane 9).

The possibility of nonsense-mediated decay occurring was eliminated by expression of Jak2 cDNAs that lacked or contained the Jak2 3’ untranslated region. The presence of the Jak2 3’ UTR resulted in comparable protein expression (Figure 1D, lanes 4 and 5). While Jak2 and Jak2K915X protein was reduced compared to the cDNA lacking the 3’ UTR (lanes 2 and 3), both forms of Jak2 were readily detected when the 3’ UTR was present.

**Figure 2:**
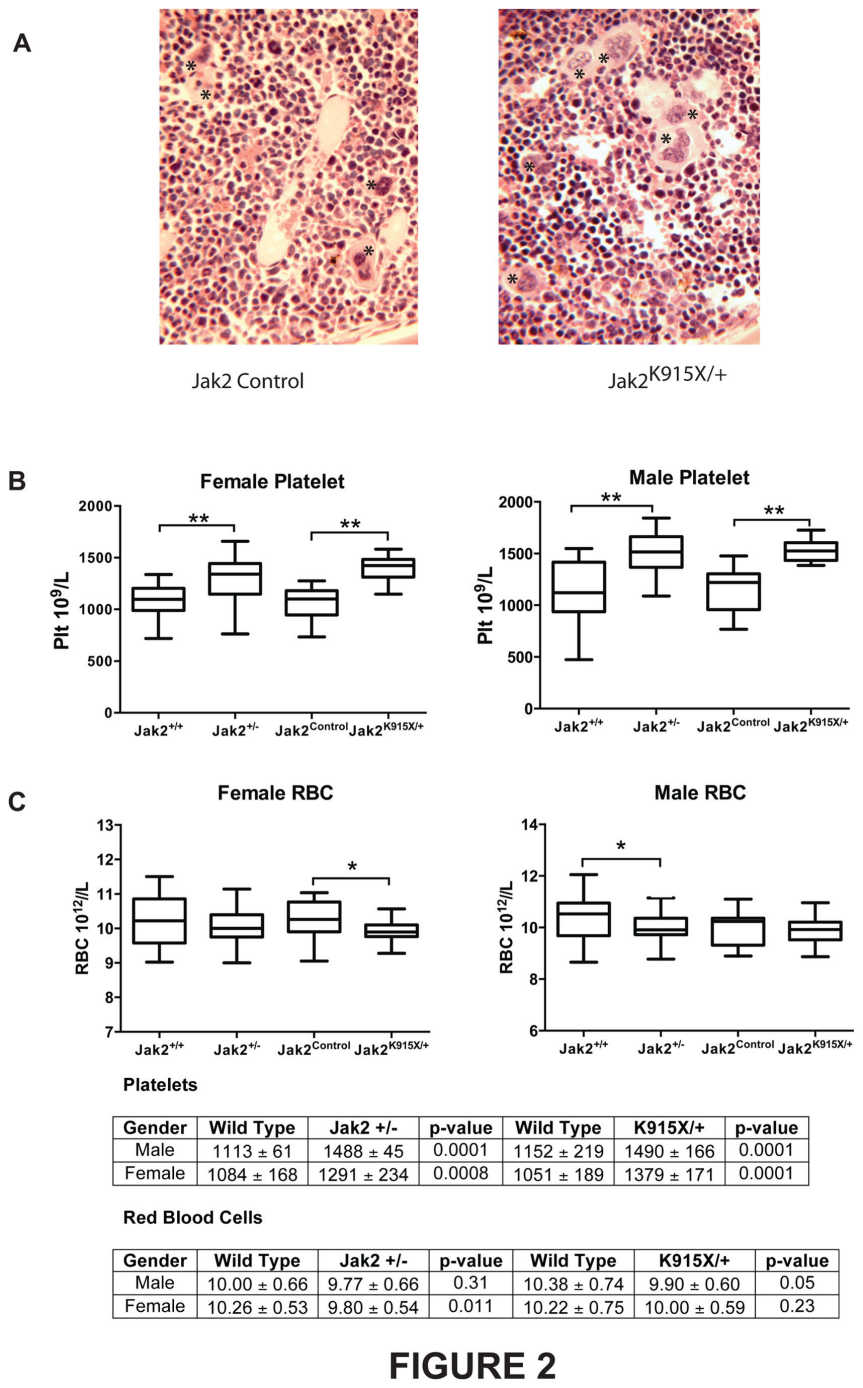
*Jak2* ^*K915X/+*^ mice have elevated megakaryocytes and platelets. (A) Bone marrow sections were prepared from 12 week *Jak2* ^*+/+*^ and *Jak2* ^*K915X/+*^ mice and stained with H and E. Representative sections are illustrated at 20x magnification. Megakaryocytes are indicated by an asterisk. (B) Platelets from male and female wild type, *Jak2* ^*+/-*^ and *Jak2* ^*K915X*^ mice at 8 wk of age were monitored. (C) Red blood cells were evaluated from male and female mice at 8 wk of age from WT, *Jak2* ^*+/-*^ and *Jak2* ^*K915X/+*^ mice. *Jak2* ^*+/+*^ or *JAK2* ^*Control*^ mice were littermate controls of *Jak2* ^*+/-*^ or *Jak2* ^*K915X/+*^ breedings, respectively. Statistically significant differences between groups are denoted as *, p< 0.05 and **, p<0.0001. Each group has n=20-30.

To determine whether the *Jak2* ^*K915X*^ allele phenocopied a Jak2 null allele or represented a neomorphic allele, we compared mice bearing homo- and heterozygous mutations in these loci. Increased megakaryocytes were observed upon enumeration of bone marrow sections isolated from *Jak2* ^*K915X/+*^ mice (Jak2^+/+^ = 7.5 ± 1.8; Jak2^K915X/+^ = 9.3 ± 2.4; p = 0.016). Representative sections from wild type and *Jak2* ^*K915X/+*^ mice illustrate increased megakaryocytes in mutant mice (Figure 2A). Elevated platelets were found in male and female mice in *Jak2* ^*K915X/+*^ mice at 8 weeks of age (Figure 2B). Interestingly, although this has not previously been reported, *Jak2* ^*+/-*^ mice showed an identical phenotype. In contrast to the platelet phenotype, red blood cell numbers (Figure 2C) and other hematological parameters (data not shown) were comparable in both *Jak2* ^*K915X/+*^ and *Jak2* ^*+/-*^ mice, with the exception of decreased RBC in *Jak2* ^*+/-*^ male and *Jak2* ^*K915X/+*^ female mice at 8 weeks, compared to *Jak2* ^*+/+*^ and *Jak2* ^*Control*^ littermates.

Clonogenic assays were performed on bone marrow and spleen cells from *Jak2* ^*K915X/+*^ and *Jak2* ^*+/-*^ mice. Interestingly, no statistically significant differences were observed in CFU-Megakaryocyte assays from bone marrow isolated from both strains of mice (Figure S1). There were no significant differences in hematopoietic progenitor number or morphology between the genotypes (Figures S1 and S2).

*Jak2* ^*K915X/+*^ and *Jak2* ^*+/-*^ mice were challenged with 120 µg/g 5-fluorouracil or 100 µg/g phenylhydrazine. Recovery curves were similar for all genotypes in response to hematopoietic stress induced by 5-fluorouracil (Figure S3) or phenylhydrazine (Figure S4).

**Figure 3:**
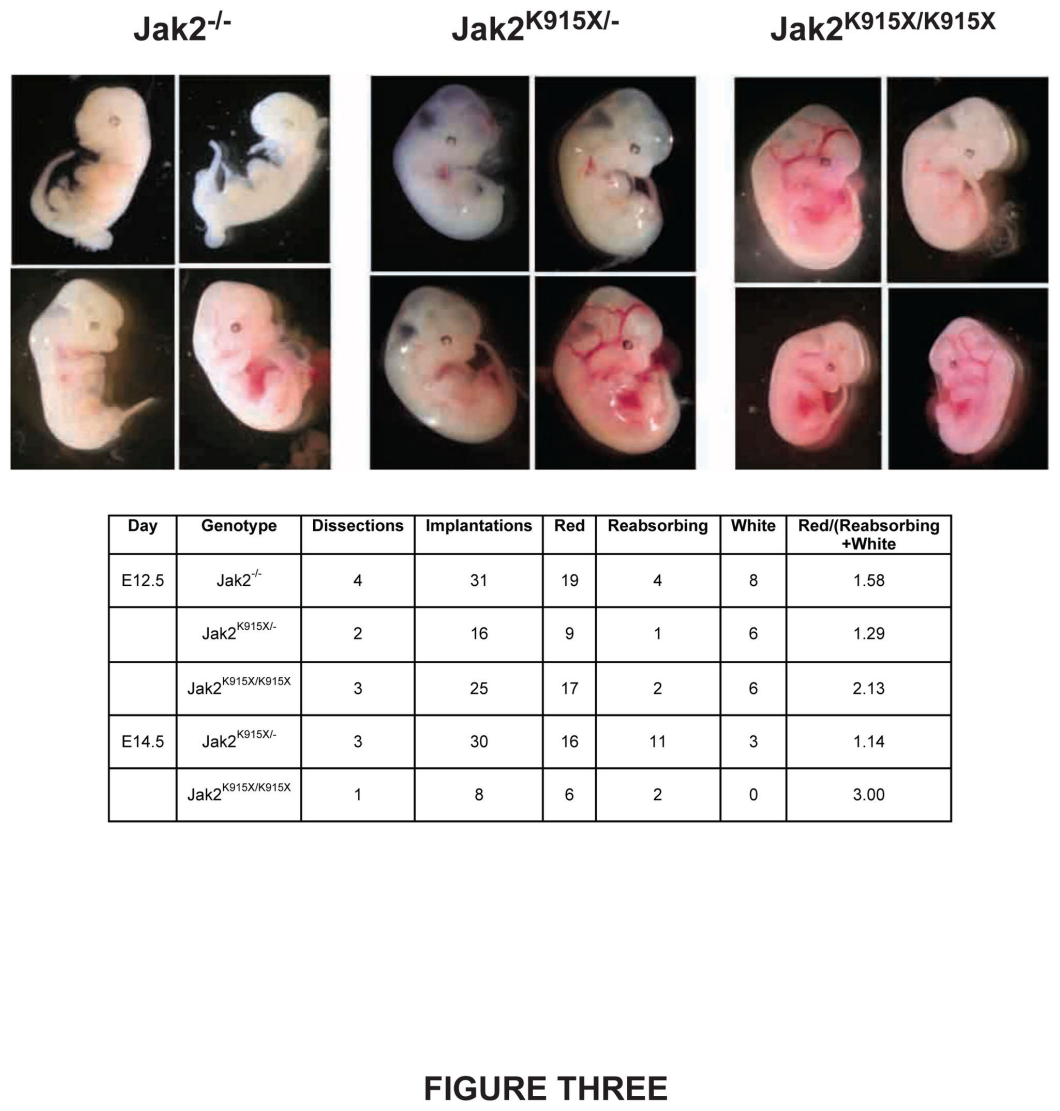
The K915X mutation in Jak2 enhances viability of mouse embryos. Embryos from timed matings were sacrificed at E12.5-E14.5. Representative embryos from E12.5 are shown. A summary of the results is provided in tabular format.

*Jak2* ^*-/-*^ embryos die at E12.5 due to a block in fetal erythropoiesis. Timed matings were conducted to generate *Jak2* ^*K915X/K915X*^ *, Jak2* ^*K915/-*^ and *Jak2* ^*-/-*^ embryos and determine whether embryonic lethality is similar between the Jak2 alleles (Figure 3). Embryos were dissected at E12.5 and E14.5 and embryos were segregated into healthy red, anemic white or terminal re-absorbing categories. Reabsorbing and white embryos were assumed to be incapable of producing viable pups. No viable *Jak2* ^*-/-*^ embryos were observed at E14.5 from three dissections comprising 26 implantations. However, viable *Jak2* ^*K915X/-*^ or *Jak2* ^*K915X/K915X*^ embryos were present at E14.5. No viable *Jak2* ^*K915X/K915X*^ embryos were identified later than E14.5.

## Discussion

The initial characterization of the Jak2 knockout mouse revealed that Jak2 played a critical role in erythropoiesis and thrombopoiesis, with embryonic lethality observed at E12.5 [14,15]. Since the EPO and EPO-R null mice die at E13.5, the slightly earlier death observed in *Jak2* ^*-/-*^ embryos was attributed to the recruitment of Jak2 to other cytokine receptors including the TPO-R. Beyond this initial evaluation of the homozygote mice, little characterization of *Jak2* ^*+/-*^ mice has been performed.

Utilizing random mutagenesis, we have demonstrated a critical, yet subtle, role for Jak2 in the regulation of megakaryopoiesis. Loss of one functional allele of *Jak2*, either through truncation in *Jak2* ^*K915X/+*^ or deletion in *Jak2* ^*+/-*^, leads to elevated platelet production. Mutation of *JAK2* is observed in several hematological disorders including ET [8,9,10,11], Polycythemia Vera [8,9,10,11], Primary Myelofibrosis [8,9,10,11] and Acute Lymphoid Leukemia (ALL) [25,26] as well as chromosomal translocations involving the fusion partners *TEL* [27,28,29], *BCR* [30], *PCM1* [31,32,33], *PAX5* [34], SEC *31A* [35] and *SSBP2* [36]. The JAK2 signaling network also participates in disease mediated by *MPL* mutations in ET and *CRLF2* mutations in T cell ALL [37,38,39,40].

The JAK2 K915X protein product does not appear to possess catalytic activity when phosphorylation of Y570 in the JH2 domain is used to monitor activity. In contrast, phosphorylation of S523 is observed in JAK2 K915X. Mutation of S523 increased catalytic activity of wild type Jak2, suggesting that S523 is a negative regulator of kinase activity [21,41]. Earlier studies suggested that phosphorylation of this residue is mediated by a proline-directed and Mek1-dependent kinase, potentially Erk [21]. Regarding the phenotype observed in *Jak2* ^*K915X/+*^ mice, both EPO [42] and TPO [43] activate Erk kinase activity and phosphorylation of Jak2 K915X could potentiate increased survival observed in timed pregnancy experiments.

Genomic resequencing efforts have identified sporadic nonsense mutations in JAK2. For example, W777X [44] and Q1112X mutations were identified in lung cancer, E890X [45] was observed in colon cancer and E1097X mutation was uncovered in a case of kidney cancer. None of these mutations were recurrent and all but the W777X mutation was confirmed. However, no further characterization of the protein products has been performed and it is unclear how these loss-of-function JAK2 mutations interact with the other genetic abnormalities observed in these patients.

Clinical trials using Jak2 inhibitors including INCB018424 [46,47], CYT387 [48] and SAR302503 (TG101380) [49] have been completed or are underway to treat primary myelofibrosis. Some patients initially responsive to JAK2 inhibition have become insensitive to JAK2 inhibitors. Whether this is due to intrinsic resistance, mutation of JAK2 [50,51,52] and its effectors, persistence due to heterodimerization with other JAK kinases [53] or other mechanisms remains to be investigated. While patients report higher quality-of-life scores, JAK2 inhibitors have not reduced allele burden, potentially due to their ability to target a spectrum of tyrosine kinases [47,54]. No studies have reported a paradoxical thrombocytosis in response to JAK2 inhibition to date. Our research suggests that altering JAK2 activation may lead to unexpected clinical outcomes.

## Supporting Information

Figure S1 **Erythroid and Megakaryocyte progenitors are unaltered in *Jak2*^*K915X/-*^and Jak2^+/-^ adult mice and do not show cytokine independent growth.** (A) CFU-MK frequency in the bone marrow grown in the presence or absence of TPO. (B) Bone marrow CFU-E frequency grown with or without EPO. (C) Splenic CFU-E frequency grown in the presence or absence of EPO. All CFU-E and CFU-Mk were derived from *Jak2* ^*K915X/-*^and Jak2^+/-^ and littermate controls at 12-14 wks of age. Data are presented as ± SEM; n=5-8.
(TIF)

Figure S2 **Functional loss of Jak2 in *Jak2*^*K915X/-*^and Jak2^+/-^ does not disrupt CFU-C frequency in the bone barrow or spleen.** (A) Total CFU-C frequency in the bone marrow. (B) The frequency of CFU-C in the spleen. (C) CFU-C differential count of bone marrow derived colonies included: CFU-G (granulocyte), CFU-M (monocyte), CFU-GEMM (granulocyte, erythrocyte, monocyte and megakaryocyte), CFU-GM (granulocyte and monocyte) and BFU-E (erythroid). (D) Splenic CFU-C differential. All CFU-C were derived from *Jak2* ^*K915X/-*^and *Jak2* ^*+/-*^ and littermate controls at 12-14wks of age. Data are presented as ± SEM; n=5-8.
(TIF)

Figure S3 **5FU hematopoietic challenge of *Jak2*^*K915X/-*^ and *Jak2*^*+/-*^ results in similar recovery.** The recovery curves for 5FU induced hematopoietic stress in *Jak2* ^*K915X/-*^ (A, C, E) and *Jak2* ^*+/-*^ (B, D, F). The recovery curves for red blood cells (A and B), platelets (C and D) and white blood cells (E and F). Data are presented as ± S.D. and n=9-11.
(TIF)

Figure S4 **PHZ challenge of erythropoiesis in *Jak2*^*K915X/-*^ and *Jak2*^+/-^.** Red blood cell recovery curves of PHZ challenged of *Jak2* ^*+/-*^ (A) and *Jak2* ^*K915X/-*^ (B). The data are presented as ± S.D.; n=8-9.
(TIF)
