# Supplementary figures and images for: Enu Mutagenesis Identifies a Novel Platelet Phenotype in a Loss-Of-Function Jak2 Allele
Source: PLoS One. 2013 Sep 25;8(9):e75472. doi: 10.1371/journal.pone.0075472 (PMC3783367; doi:10.1371/journal.pone.0075472)

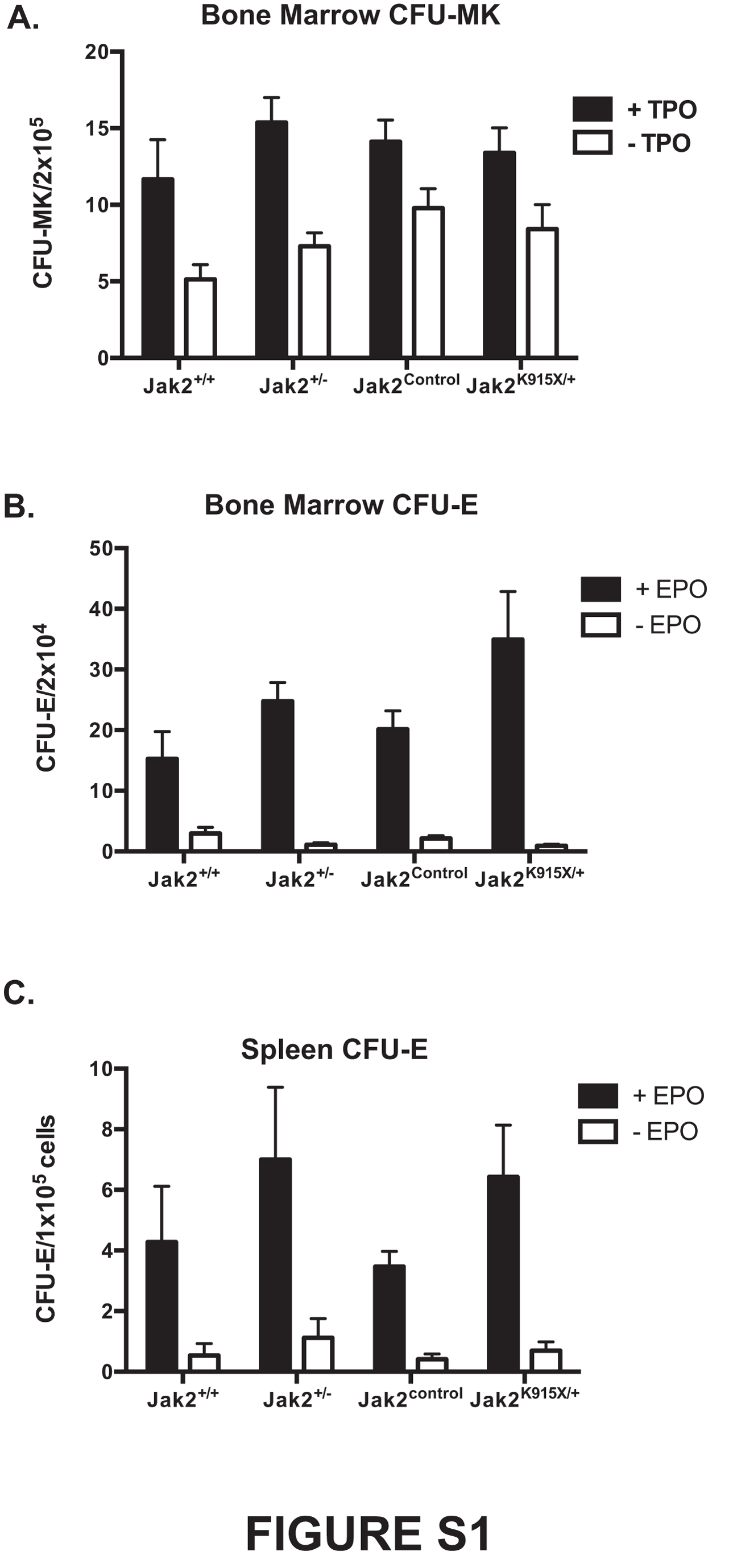

Supplement: Figure S1 — Erythroid and Megakaryocyte progenitors are unaltered in Jak2K915X/-and Jak2+/- adult mice and do not show cytokine independent growth. (A) CFU-MK frequency in the bone marrow grown in the presence or absence of TPO. (B) Bone marrow CFU-E frequency grown with or without EPO. (C) Splenic CFU-E frequency grown in the presence or absence of EPO. All CFU-E and CFU-Mk were derived from Jak2 K915X/-and Jak2+/- and littermate controls at 12-14 wks of age. Data are presented as ± SEM; n=5-8. (TIF) [file pone.0075472.s001.tif]

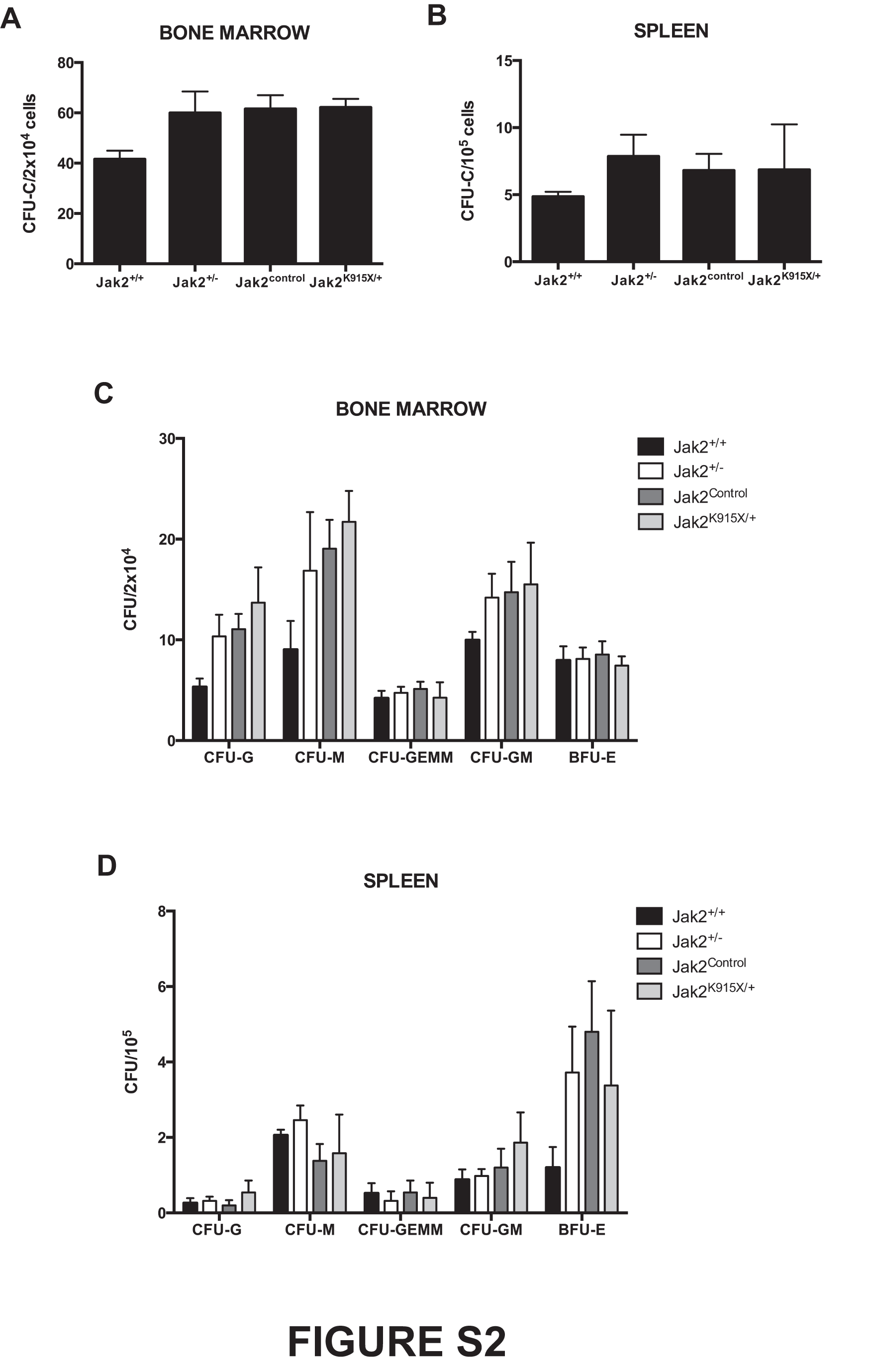

Supplement: Figure S2 — Functional loss of Jak2 in Jak2K915X/-and Jak2+/- does not disrupt CFU-C frequency in the bone barrow or spleen. (A) Total CFU-C frequency in the bone marrow. (B) The frequency of CFU-C in the spleen. (C) CFU-C differential count of bone marrow derived colonies included: CFU-G (granulocyte), CFU-M (monocyte), CFU-GEMM (granulocyte, erythrocyte, monocyte and megakaryocyte), CFU-GM (granulocyte and monocyte) and BFU-E (erythroid). (D) Splenic CFU-C differential. All CFU-C were derived from Jak2 K915X/-and Jak2 +/- and littermate controls at 12-14wks of age. Data are presented as ± SEM; n=5-8. (TIF) [file pone.0075472.s002.tif]

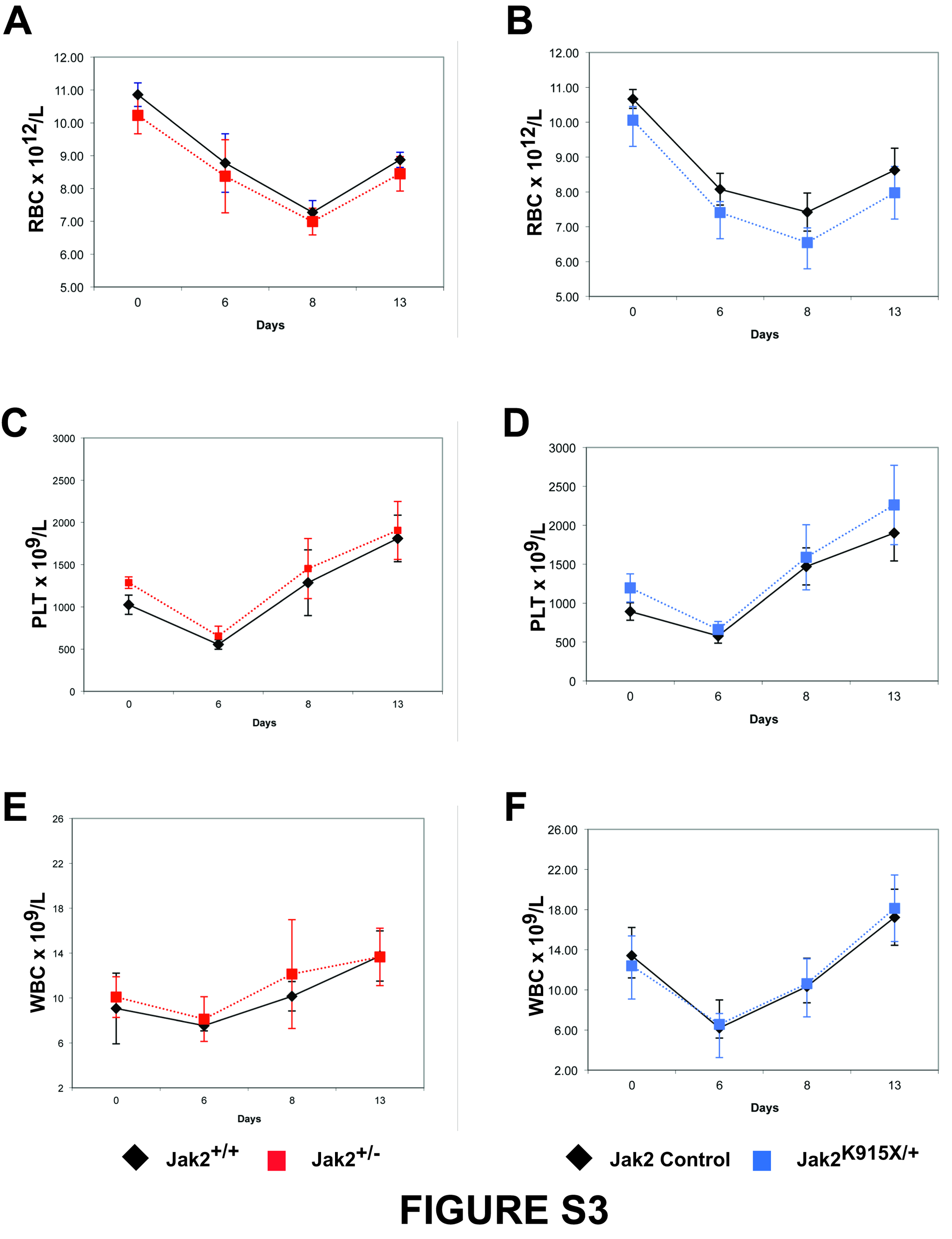

Supplement: Figure S3 — 5FU hematopoietic challenge of Jak2K915X/- and Jak2+/- results in similar recovery. The recovery curves for 5FU induced hematopoietic stress in Jak2 K915X/- (A, C, E) and Jak2 +/- (B, D, F). The recovery curves for red blood cells (A and B), platelets (C and D) and white blood cells (E and F). Data are presented as ± S.D. and n=9-11. (TIF) [file pone.0075472.s003.tif]

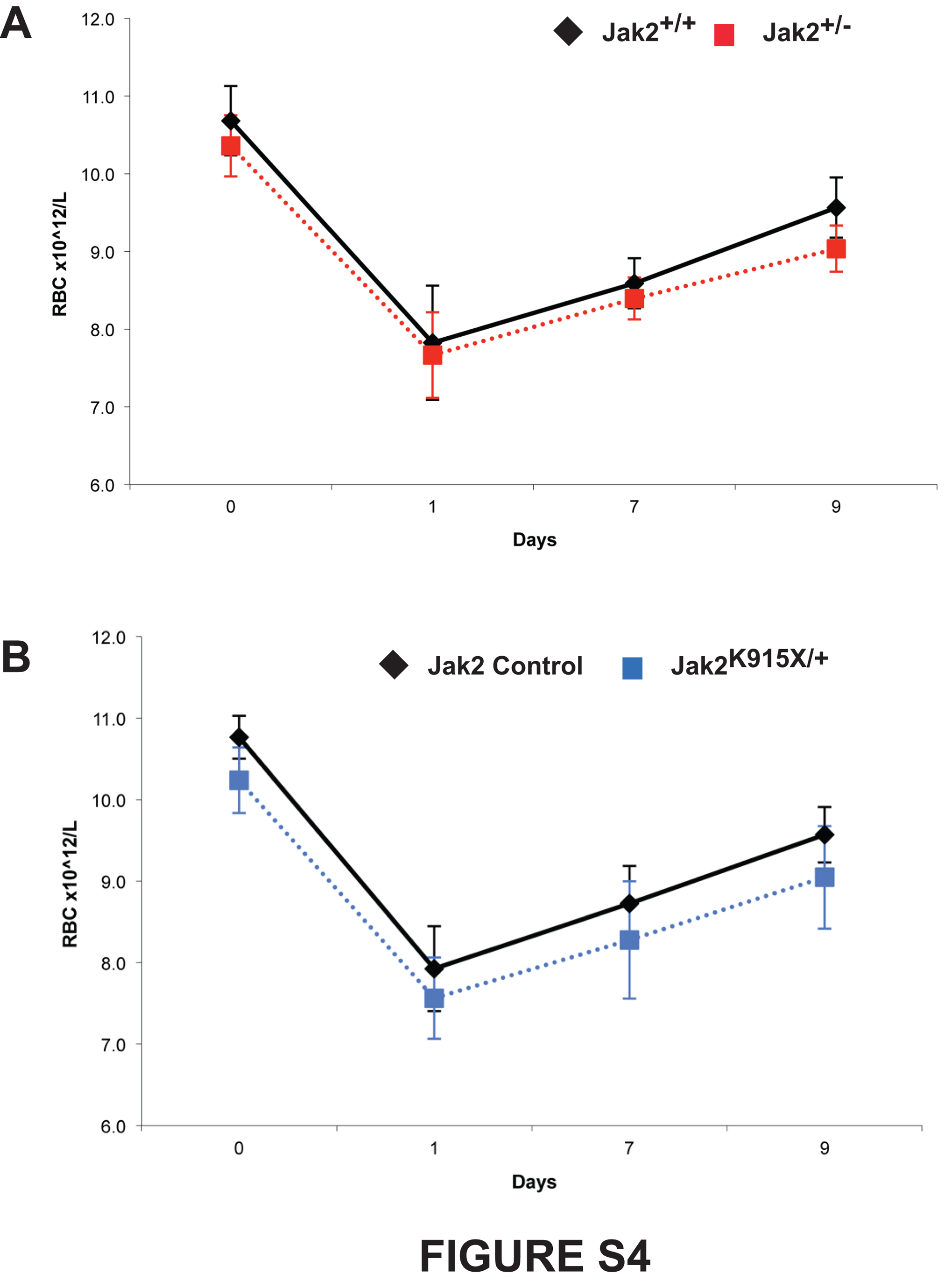

Supplement: Figure S4 — PHZ challenge of erythropoiesis in Jak2K915X/- and Jak2+/-. Red blood cell recovery curves of PHZ challenged of Jak2 +/- (A) and Jak2 K915X/- (B). The data are presented as ± S.D.; n=8-9. (TIF) [file pone.0075472.s004.tif]
